# Supplementary material for: Sinding-Larsen-Johansson disease. Clinical features, imaging findings, conservative treatments and research perspectives: a scoping review
Source: PeerJ. 2024 Sep 25;12:e17996. doi: 10.7717/peerj.17996 (PMC11438430; doi:10.7717/peerj.17996)
Supplement: Supplemental Information 1 [file peerj-12-17996-s001.docx]

# Table S1. Quality of the included studies – JBI Critical Appraisal for Case Reports

| No. | Authors | Patient’s demographic characteristics clearly described? | Patient’s history clearly described and presented as a timeline? | Current clinical condition of the patient on presentation clearly described? | Diagnostic tests or assessment methods and the results clearly described? | Intervention(s) or treatment procedure(s) clearly described? | Post-intervention clinical condition clearly described? | Adverse events (harms) or unanticipated events identified and described? | Case report provides takeaway lessons? | Scores (8) | Quality |
| --- | --- | --- | --- | --- | --- | --- | --- | --- | --- | --- | --- |
| 1 | Valentino et al., 2012 | Yes | Yes | Yes | Yes | Yes | Yes | NA | Yes | 7 | Low risk of bias |
| 2 | Davis et al., 2010 | Yes | No | Yes | Yes | No | No | NA | Yes | 4 | Moderate risk of bias |
| 3 | De Flaviis, 1989 | Yes | No | Yes | Yes | No | No | NA | Yes | 4 | Moderate risk of bias |
| 4 | Dupuis et al., 2009 | Yes | No | Yes | Yes | No | No | NA | Yes | 4 | Moderate risk of bias |
| 5 | Franceschi, 2006 | Yes | Yes | Yes | Yes | Yes | Yes | NA | Yes | 7 | Low risk of bias |
| 6 | KUENHAST 2012 | Yes | Yes | Yes | Yes | Yes | Yes | NA | Yes | 7 | Low risk of bias |
| 7 | Malherbe 2012 | Yes | No | Yes | Yes | No | No | NA | Yes | 4 | Moderate risk of bias |
| 8 | Iwamoto 2009 | Yes | No | Yes | Yes | No | No | NA | Yes | 4 | Moderate risk of bias |
| 9 | Goldmann et al. 2009 | Yes | Yes | Yes | Yes | No | No | NA | Yes | 5 | Moderate risk of bias |
| 10 | Tourdias 2015 | Yes | Yes | Yes | Yes | Yes | Yes | NA | Yes | 7 | Low risk of bias |
| 11 | Bonney 1948 | Yes | Yes | Yes | Yes | Yes | Yes | NA | Yes | 7 | Low risk of bias |
| 12 | Carr 2001 | Yes | No | Yes | Yes | No | No | NA | Yes | 4 | Moderate risk of bias |
| 13 | Alito et al. 2023 | Yes | Yes | Yes | Yes | Yes | Yes | NA | Yes | 7 | Low risk of bias |
| 14 | Sinding-Larsen 1921 | Yes | Yes | Yes | Yes | Yes | Yes | NA | Yes | 7 | Low risk of bias |
