# Supplementary material for: Sinding-Larsen-Johansson disease. Clinical features, imaging findings, conservative treatments and research perspectives: a scoping review
Source: PeerJ. 2024 Sep 25;12:e17996. doi: 10.7717/peerj.17996 (PMC11438430; doi:10.7717/peerj.17996)
Supplement: Supplemental Information 2 [file peerj-12-17996-s002.docx]

**Systematic review and/or meta-analyses rationale and contribution**

**The rationale for conducting the meta-analysis**

The rationale for conducting this scoping review arises from the need to systematically address several gaps in the existing literature on Sinding-Larsen-Johansson Disease (SLJD). Despite the clinical significance of SLJD in physically active adolescents, the current body of evidence is predominantly limited to case studies, leading to fragmented and often conflicting data. This lack of comprehensive analysis hinders the establishment of standardized diagnostic and treatment protocols. Furthermore, the overlapping symptoms of SLJD with other knee pathologies complicate accurate diagnosis, necessitating a thorough synthesis of available diagnostic tools and clinical features. The growing prevalence of sports specialization and the corresponding increase in sports-related injuries among adolescents highlight the urgency of understanding SLJD within the context of modern sports medicine. Therefore, this scoping review aims to consolidate existing research on the pathogenesis, clinical diagnosis, imaging outcomes, and conservative treatments of SLJD, thereby providing a clearer understanding of the disease and identifying areas requiring further investigation.

**The contribution that the meta-analysis makes to knowledge in light of previously published related reports, including other meta-analyses and systematic reviews.**

This scoping review makes a significant contribution to the existing knowledge of SLJD by systematically consolidating and synthesizing the available evidence, which has hitherto been scattered and inconsistent across individual case reports. Previous reports and reviews have primarily focused on broader categories of knee pathologies or similar conditions, such as Osgood-Schlatter disease, often mentioning SLJD only in passing without detailed examination. By focusing specifically on SLJD, this review highlights its unique clinical features, diagnostic challenges, and treatment outcomes, filling a crucial gap in the literature.

The review's methodology, adhering to the PRISMA guidelines for scoping reviews, ensures a comprehensive and systematic approach to literature search and data synthesis. By utilizing multiple databases, including PubMed, Scopus, Medline OVID, Embase, Web of Science, and Grey literature, this review captures a wide array of studies, enhancing the breadth and depth of the analysis. The use of the Joanna Briggs Institute (JBI) checklist for quality assessment further reinforces the rigor and reliability of the findings.

In light of previously published reports, this scoping review serves as a critical bridge between disparate case studies, providing a consolidated evidence base that can inform clinical practice and guide future research. It underscores the necessity for more extensive cohort studies and clinical trials to refine the management and treatment protocols for SLJD. Additionally, this review addresses the need for education and modification of physical activity among young athletes, aligning with contemporary trends in sports medicine and injury prevention.

Overall, this scoping review not only enhances the understanding of SLJD but also lays the groundwork for future research initiatives aimed at improving diagnostic accuracy and treatment outcomes for affected adolescents.
